# Supplementary material for: Young children do not require perceptual-motor feedback to solve Aesop’s Fable tasks
Source: PeerJ. 2017 Jul 17;5:e3484. doi: 10.7717/peerj.3484 (PMC5516770; doi:10.7717/peerj.3484)
Supplement: Table S1 — P-values (‘p’) are calculated from exact two-tailed binomial tests. Significant p-values are highlighted in bold. [file peerj-05-3484-s001.docx]

| **Condition** | **Across all trials % correct** | ***p*-value** |
| --- | --- | --- |
| Large vs. small | 73 | **<0.0001** |
| Too large vs. small | 74 | **<0.0001** |
| Floating vs. sinking | 82 | **<0.0001** |
| Hollow vs. solid | 75 | **<0.0001** |
| Wide vs. narrow | 50 | >0.999 |
| High vs. low | 86 | **<0.001** |

**Table S1.** Correct choices (%) in each condition across all subjects (n=55). *P*-values (‘*p*’) are calculated from exact two-tailed binomial tests. Significant p-values are highlighted in bold.
