# Supplementary material for: Young children do not require perceptual-motor feedback to solve Aesop’s Fable tasks
Source: PeerJ. 2017 Jul 17;5:e3484. doi: 10.7717/peerj.3484 (PMC5516770; doi:10.7717/peerj.3484)
Supplement: Table S2 — P-values (‘p’) are calculated from exact two-tailed binomial tests. Significant p-values are highlighted in bold. NS, not significant with a Bonferroni correction. [file peerj-05-3484-s002.docx]

| **Age in years** | **Large vs. small** | | **Too large vs. small** | | **Floating vs. sinking** | | **Hollow vs. solid** | | **Wide vs. narrow** | | | **High vs. low** | |
| --- | --- | --- | --- | --- | --- | --- | --- | --- | --- | --- | --- | --- | --- |
|  | % | *p* | % | *p* | % | *p* | % | *p* | % | *p* | | % | *p* |
| **Trial 1** | | | | | | | | | | | | | |
| 5 | 60 | 0.75 | 30 | 0.34 | 30 | 0.34 | 20 | 0.11 | 30 | 0.34 | | 100 | **0.002** |
| 6 | 85 | *0.023 NS* | 23 | 0.09 | 54 | >0.99 | 54 | >0.99 | 31 | 0.27 | | 77 | 0.09 |
| 7 | 82 | 0.065 | 36 | 0.55 | 82 | 0.065 | 82 | 0.065 | 55 | >0.99 | | 64 | 0.55 |
| 8 | 100 | **0.001** | 27 | 0.23 | 91 | *0.012 NS* | 91 | *0.012 NS* | 55 | >0.99 | | 91 | *0.012 NS* |
| 9 | 90 | *0.022 NS* | 60 | *0.022 NS* | 90 | *0.022 NS* | 80 | 0.12 | 70 | 0.34 | | 70 | 0.34 |
| **Across all trials** | | | | | | | | | | | | | |
| 5 | 46 | 0.67 | 34 | *0.015 NS* | 60 | 0.2 | 48 | 0.887 | 20 | | 0.3 | 88 | **<0.0001** |
| 6 | 69 | **0.003** | 69 | **0.003** | 71 | **0.001** | 71 | **0.001** | 34 | | *0.013 NS* | 92 | **<0.0001** |
| 7 | 69 | **0.007** | 75 | **0.0004** | 93 | **<0.0001** | 85 | **<0.0001** | 67 | | *0.014 NS* | 75 | **0.0004** |
| 8 | 91 | **<0.0001** | 76 | **0.0001** | 96 | **<0.0001** | 85 | **<0.0001** | 65 | | *0.03 NS* | 93 | **<0.0001** |
| 9 | 92 | **<0.0001** | 84 | **<0.0001** | 92 | **<0.0001** | 86 | **<0.0001** | 64 | | 0.065 | 80 | **<0.0001** |

**Table S2.** Correct choices (%) in each condition per year group. *P*-values (‘*p*’) are calculated from exact two-tailed binomial tests. Significant *p*-values are highlighted in bold. *NS* = not significant with a Bonferroni correction.
